# Adding 5-hydroxytryptamine receptor type 3 antagonists may reduce drug-induced nausea in poor insight obsessive-compulsive patients taking off-label doses of selective serotonin reuptake inhibitors: a 52-week follow-up case report

**DOI:** 10.1186/1744-859X-9-39

**Published:** 2010-12-10

**Authors:** Michele Fornaro, Matteo Martino

**Affiliations:** 1 Department of Psychiatry, University of Genova, Genoa, Italy

## Abstract

Poor-insight obsessive-compulsive disorder (PI-OCD) is a severe form of OCD where the 'typically obsessive' features of intrusive, 'egodystonic' feelings and thoughts are absent. PI-OCD is difficult to treat, often requiring very high doses of serotonergic drugs as well as antipsychotic augmentation. When this occurs, unpleasant side effects as nausea are common, eventually further reducing compliance to medication and increasing the need for pharmacological alternatives. We present the case of a PI-OCD patient who developed severe nausea after response to off-label doses of the selective serotonin reuptake inhibitor (SSRI), fluoxetine. Drug choices are discussed, providing pharmacodynamic rationales and hypotheses along with reports of rating scale scores, administered within a follow-up period of 52 weeks. A slight reduction of fluoxetine dose, augmentation with mirtazapine and a switch from amisulpride to olanzapine led to resolution of nausea while preserving the anti-OCD therapeutic effect. Mirtazapine and olanzapine have already been suggested for OCD treatment, although a lack of evidence exists about their role in the course of PI-OCD. Both mirtazapine and olanzapine also act as 5-hydroxytryptamine receptor type 3 (5-HT3) blockers, making them preferred choices especially in cases of drug-induced nausea.

## Background

Poor-insight obsessive-compulsive disorder (PI-OCD) is an unusual condition where the 'typically obsessive' features of intrusive, 'egodystonic' feelings and thoughts are absent and the course and severity of the illness are usually more severe than that seen with the classical, egodystonic form of the disorder [1]. Remarkably, PI-OCD often requires higher therapeutic doses of serotonergic drugs, even beyond in-label ranges, than classical OCD, frequently requiring augmentation strategies with antipsychotic drugs [2]. Consequently, the high postsynaptic 5-hydroxytryptamine receptor type 2A (5-HT2A) stimulation of the gastrointestinal tract due to a consistent dose of the serotonergic drug can lead to impairing side effects, including nausea [3], which may in turn account for some of the cases of discontinuation of treatment [4].

## Case presentation

Our patient is a 26-year-old Caucasian man with severe PI-OCD, first diagnosed at age of 22 and unresponsive to repetitive 'adequate' [5] trials of selective serotonin reuptake inhibitors (SSRIs), tricyclic antidepressants (TCAs) and/or antipsychotic medications, as well as cognitive and behavioural psychotherapy (CBT). From ages 18 to 22, the patient was treated by a psychiatrist with alternative trials of SSRIs, including paroxetine 50 mg/day and sertraline 200 mg/day. TCAs such as clomipramine 300 mg/day plus perphenazine 4 mg/day and biperidon 4 mg/day were also prescribed for the presence of partial critical obsessions regarding his physical appearance. All these pharmacological trials lasted for at least 6 months each, but none of them lead to a substantial improvement of his clinical picture, providing just partial resolution of illness. During that period of time, his compliance with medication was poor, with the role of his parents being essential in guaranteeing regular medical follow-up. He finally stopped his medication at age 22 when he moved out of his home town in search of work. Anamnestic information about the patient from ages 22 to 26 was considered unreliable, as it relied on his own controversial assertions. Indeed, his lack of insight of illness probably led to complete withdrawal of medication during that period of time and worsening of illness with progressive reduction of insight. When he eventually returned to his home town at the age of 26, after spontaneous departure from his occupation, the patient returned to his parent's house. The patient said he left his work because of his intense fear of being unable to 'control' his own eyes 'smiling' in public. Although his insight of illness was almost absent, the patient accepted the request of his parents for a clinical evaluation by a new psychiatrist. When admitted to our outpatient facility, the same obsessions were evident. Apparently, the patient had no other obsessions or compulsions.

According to the Diagnostic and Statistical Manual of Mental Disorders, fourth edition (DSM-IV) criteria assessed by administration of the Structured Clinical Interview of Axis-I and Axis-II Disorder (SCID-I and SCID-II) [6,7], he had no other relevant psychiatric comorbidity other than PI-OCD and dysthymia. His lifetime medical history was also negative. Remarkably, according to available information provided by the patient himself and by reports from significant others (his relatives), he had no premorbid personality disorder or temperamental history. Insight into his illness and therefore his compliance to therapy was poor. In such scenarios, obtaining the patient's compliance to medication is as important as it is difficult to achieve, requiring continuative familial support. The clinical impressions were confirmed by the high total scores the patient obtained on the Yale Brown Obsessive-Compulsive Scale (Y-BOCS) [8] and at the Brown Assessment of Beliefs Scale (BABS) [9], when the SSRI fluoxetine 90 mg/day (an off-label drug administered at 40 mg in the morning and 50 mg in the evening) and the atypical antipsychotic amisulpride 600 mg/day (divided into two administrations a day) were prescribed together with diazepam 5 mg/day (twice a day).

## Results

**Figure 1:**
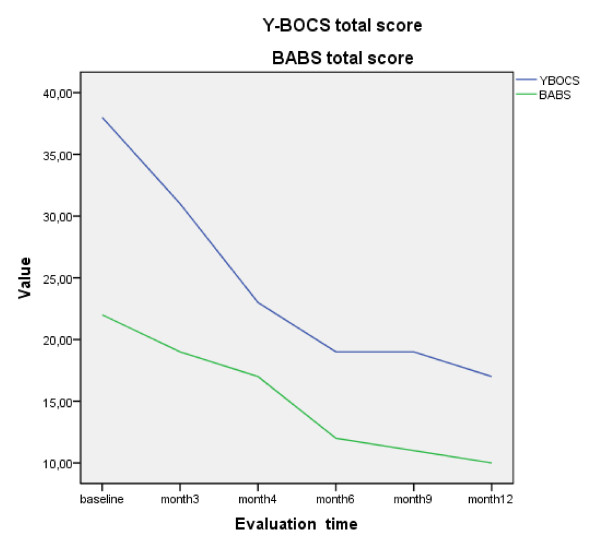
**Trend of Yale Brown Obsessive-Compulsive Scale (Y-BOCS) and Brown Assessment of Beliefs Scale (BABS) total score within 1-year follow-up**. First 3 months of therapy refer to 90 mg/day fluoxetine and 600 mg/day amisulpride, while following observations refer to 60 mg/day fluoxetine plus 30 mg/day mirtazapine and 10 mg/day olanzapine.

The Y-BOCS and the BABS scales showed total scores of 38 and 22, respectively, (with a score of 4 for the item 'conviction' and 4 for the item 'insight' on the BABS); on the BABS scale, 4 is the highest possible score and it indicates worse symptomatology [9] while Y-BOCS score ranges are considered as follows: 0-7 subclinical, 8-15 mild, 16-23 moderate, 24-32 severe and 32-40 extreme. A clinical follow-up was obtained after 3 months. The patient was asked to answer the same rating scales again. The new Y-BOCS total score was 31 while the BABS total score was recorded at 19 ('conviction' and 'insight' items were both scored as 4). Clinical improvement was seen, although the patient still showed reluctance toward pharmacotherapy, essentially because of the presence of severe nausea. Despite the difficulty in obtaining an appreciable clinical response and acceptable insight of illness, the therapeutic scheme was revised in order to relieve nausea, which seriously undermined the already weak adherence of the patient to treatment. The Patient Rated Inventory of Side Effects (PRISE; developed for the National Institute of Mental Health (NIMH)-funded Sequenced Treatment Alternatives to Relieve Depression (STAR*D) protocol) [10] was also administered to assess the impact of side effects in the course of therapy, highlighting a 'distressing' level of nausea. The fluoxetine dose was reduced to 60 mg/day, adding 30 mg/day of mirtazapine and 10 mg/day of olanzapine instead of amisulpride 600 mg/day. Diazepam was discontinued due to excessive sedation of the patient. Nausea remitted after just 2 weeks of initiation of the new therapy. During follow-up visits at months 4 (when the same kind of obsessions were still present, but at a less intrusive extent), 6, 9 and 12 (final observation) substantial maintenance of antiobsessive efficacy and increase of insight of illness were clinically observed and confirmed by rating evaluations (Table 1 and Figure 1).

**Table 1:** Yale Brown Obsessive-Compulsive Scale (Y-BOCS) and Brown Assessment of Beliefs Scale (BABS) total score assessed within 1 year of follow-up

Evaluation time	Y-BOCS total score	BABS total score
Baseline	38.00	22.00
Month 3	31.00	19.00
Month 4	23.00	17.00
Month 6	19.00	12.00
Month 9	19.00	11.00
Month 12	17.00	10.00

First 3 months of therapy refer to 90 mg/day fluoxetine and 600 mg/day amisulpride, while following observations refer to 60 mg/day fluoxetine plus 30 mg/day mirtazapine and 10 mg/day olanzapine.

Although not impressive, the 1-year treatment outcome was considered satisfactory for a PI-OCD case, usually characterised by mild response to medications and requirement for long-term therapy.

## Discussion

The poor-insight feature of PI-OCD makes it a difficult condition for therapists. Obsessions and delusions have been traditionally viewed as dichotomous phenomena, with obsessions defined as 'intrusive, egodystonic thoughts with the patient maintaining insight'. By contrast, delusions have been defined as false beliefs firmly held by the patient without insight into the irrationality of the belief. However, obsessions and delusions may be better conceptualised as existing on a continuum of insight that ranges from good insight (overvalued ideation, as in typical OCD) to poor insight/no insight (delusional thinking) as in the course of PI-OCD [9]. When 'psychotic features' are present in the course of OCD, treatment is often difficult, requiring high doses of standard OCD medication and frequently requiring augmentation strategies with both proserotonergic drugs and antipsychotics, especially second generation compounds associated with more favourable general side-effect profiles [11]. As a consequence, such high doses of drugs (often beyond in-label ranges), can be associated with unpleasant side effects, including nausea as one the most frequent occurrences [12]. Furthermore, no univocal pharmacological management of PI-OCD and potential iatrogenic effects exists. This is essentially due to a relatively low prevalence [2] of the disorder and difficulty in detection of related clinical features.

Fluoxetine (in-label doses for depression and anxiety disorders range between 20-80 mg/day) is an SSRI antidepressant stimulating 5-HT2A postsynaptic receptors and desensitising 1A receptors; it also acts as a 5-HT2C antagonist, increasing norepinephrine and dopamine neurotransmission at the prefrontal cortex [13]. Both 5-HT2A and 5-HT2C actions may play a major role in its antiobsessive actions, although 5-HT2A stimulation can induce nausea, especially at higher doses [13], conversely reducing striatal dopaminergic neurotransmission [2].

Amisulpride is an atypical antipsychotic possibly acting as a dopamine stabiliser and dopamine partial agonist. Although not a first-line choice antipsychotic augmentation strategy for PI-OCD cases unresponsive to serotonergic monotherapy, it may be considered when dysthymia is also present, as it was in our case. While low doses (for example, 50 mg/day) of amisulpride may be suggested for dysthymia (disinhibiting presynaptic D2 receptors), higher doses (blocking postsynaptic D2 receptors) may be required in case of negative symptomatology (50-300 mg/day) or positive symptoms (400-800 mg/day for schizophrenia). Additionally, the gastrointestinal D2 blockade may contribute to an antiemetic effect of amisulpride or other benzamides (for example, metoclopramide) with potential gastroprokinetics actions at high doses (possibly also from 5-HT3 receptor antagonism).

Nonetheless, as apparently occurred in our case, 5-HT2A stimulation may prevail on the atypical antipsychotic '5-HT2A > D2' blockade, inducing nausea eventually unresponsive to standard antiemetic medications. In such cases, a slight reduction of the SSRI dose may be made, suggesting an add-on therapy with a second antidepressant medication as the α2 antagonist, dual serotonin and norepinephrine agent (NaSSA) mirtazapine (for example, at 30 mg/day) [14], which may help in anti-obsessive and antiemetic management via 5-HT3 antagonism and 5-HT2A blockade [13,15]. Switching amisulpride to olanzapine (for example, at 10 mg/day) may also help since the antipsychotic action may be coupled to a strong 5-HT3 blockade, whereas olanzapine 5-HT2C blockade should be an optimal complement to fluoxetine in the management of affective, anxious and cognitive symptoms [13].

Interestingly, blocking the ligand-gated ion channel 5-HT3 receptor [13] may not only exert an antiemetic effect but also a putative intrinsic antiobsessive action, as suggested by the efficacy of ondansetron (a potent 5-HT3 antagonist used for chemotherapy-induced nausea) augmentation in treatment-resistant OCD [16,17], whereas olanzapine and mirtazapine 5-HT3 blockade has been considered for cancer chemotherapy-related nausea and cachexia (due to the H1 blockade) [18].

Indeed, both compliance issues related to the total number of medications and anti-OCD therapeutic implications should not support the choice of non-psychiatric 5-HT3 blockers in favour of proven anti-OCD psychopharmacological agents sharing the 5-HT3 antagonist property. Finally, while more research is needed in this direction, mirtazapine and olanzapine could play a major role in PI-OCD augmentation strategies for patients taking high (sometimes off-label) doses of SSRIs, and their 5-HT3 blockade may substantially contribute to the management of drug-induced nausea, even if as in this case it remains unclear which one of the two medications might have had most contribution to cessation of nausea or contribution to compliance lasting for at least 1 year.

## Conclusions

In this report, the case of a PI-OCD patient responding to off-label doses of SSRIs, which induced nausea potentially precluding the therapeutic compliance and the final outcome, is presented. Addition of 5-HT3 serotonergic antagonists led to a still-effective management of PI-OCD and substantial resolution of nausea, which lasted for up to 1 year of follow-up.

## Competing interests

The authors declare that they have no competing interests.

## Authors' contributions

MF designed the study and wrote the main text. MM contributed to the drafting of the manuscript and rating evaluation within the follow-up period. Both authors read and approved the final manuscript.

## Consent

Written informed consent was obtained from the patient for publication of this case report. A copy of the written consent is available for review by the Editor-in-Chief of this Journal.
